# Cervical Granulocytic Sarcoma Without Acute Leukemia: A Case Report and Review of 42 Additional Cases

**DOI:** 10.7759/cureus.72341

**Published:** 2024-10-24

**Authors:** Wenting Zhu, Chengbei Tao, Zhengying Ruan, Linglong Xu

**Affiliations:** 1 School of Medicine, Shaoxing University, Shaoxing, CHN; 2 Department of Urology Surgery, The First People's Hospital of Wenling, Taizhou, CHN; 3 Department of Pathology, Taizhou Central Hospital (Taizhou University Hospital), Taizhou, CHN; 4 Department of Hematology, Taizhou Central Hospital (Taizhou University Hospital), Taizhou, CHN

**Keywords:** acute myeloid leukemia (aml), acute myeloid sarcoma, chemotherapy, granulocytic sarcoma, myeloid sarcoma

## Abstract

Granulocytic sarcoma (GS), also known as extramedullary myeloid tumor, is a rare malignant neoplasm composed of immature myeloid cells. Although it is most commonly associated with acute myeloid leukemia (AML), a subset of GS cases can occur prior to the development of AML. GS can present in a variety of extramedullary locations, including bone, skin, lymph nodes, and the female reproductive system. We report the case of a 45-year-old Asian female patient who presented to our hospital in July 2015 with a three-month history of left-sided lumbago. A contrast-enhanced computed tomography (CT) scan of the abdomen and pelvis revealed a retroperitoneal mass and an enlarged uterine cervix with an accompanying 57×52 mm mass. A cervical biopsy confirmed the diagnosis of GS. Immunohistochemical (IHC) analysis of the biopsy showed that the neoplastic cells were positive for CD34, CD15, CD33, CD43, lysozyme, and myeloperoxidase. The patient was subsequently treated with an idarubicin and cytarabine-based regimen for four cycles. A follow-up CT scan of the abdomen and pelvis demonstrated a significant reduction in the size of the previous lesions. Unfortunately, the patient passed away in April 2016 due to a cerebral hemorrhage. In this report, we also review 42 additional cases to discuss the pathological characteristics, treatment strategies, and clinical outcomes of GS.

## Introduction

Granulocytic sarcoma (GS), also known as extramedullary myeloid tumor, is a rare malignant neoplasm characterized by the proliferation of immature myeloid cells [1,2]. While it typically occurs in association with acute myeloid leukemia (AML), a subset of GS cases presents before the onset of AML [3]. GS can arise in a range of extramedullary locations, including bone, skin, lymph nodes, and the female reproductive system [4]. Among these, the ovaries are the most frequently affected, followed by the cervix and uterus [5]. This article presents a systematic review of GS cases involving the cervix, along with a case report of GS exhibiting an atypical clinical presentation in the absence of concurrent acute leukemia [6].

## Case presentation

A 45-year-old Asian female patient was referred to our hospital in July 2015, presenting with a three-month history of left-sided lumbago. She had a 10-year history of hypertension, managed with oral enalapril maleate (one tablet daily) and indapamide (one tablet daily), with good blood pressure control. Additionally, she had a six-month history of diabetes, treated with gliclazide (one tablet twice daily), though her blood glucose control was unclear. Over the past six months, she also reported irregular menstruation, with increased flow and blood clots, though the color of the blood remained normal. Her medical and family history otherwise remained unremarkable. She had no known family history of malignancy. The patient was married at the age of 20 and had two healthy children. Her parents, one brother, and one sister were all in good health. On physical examination, the only notable findings were tenderness on percussion in the left kidney area and the presence of a cervical mass.

**Figure 1:**
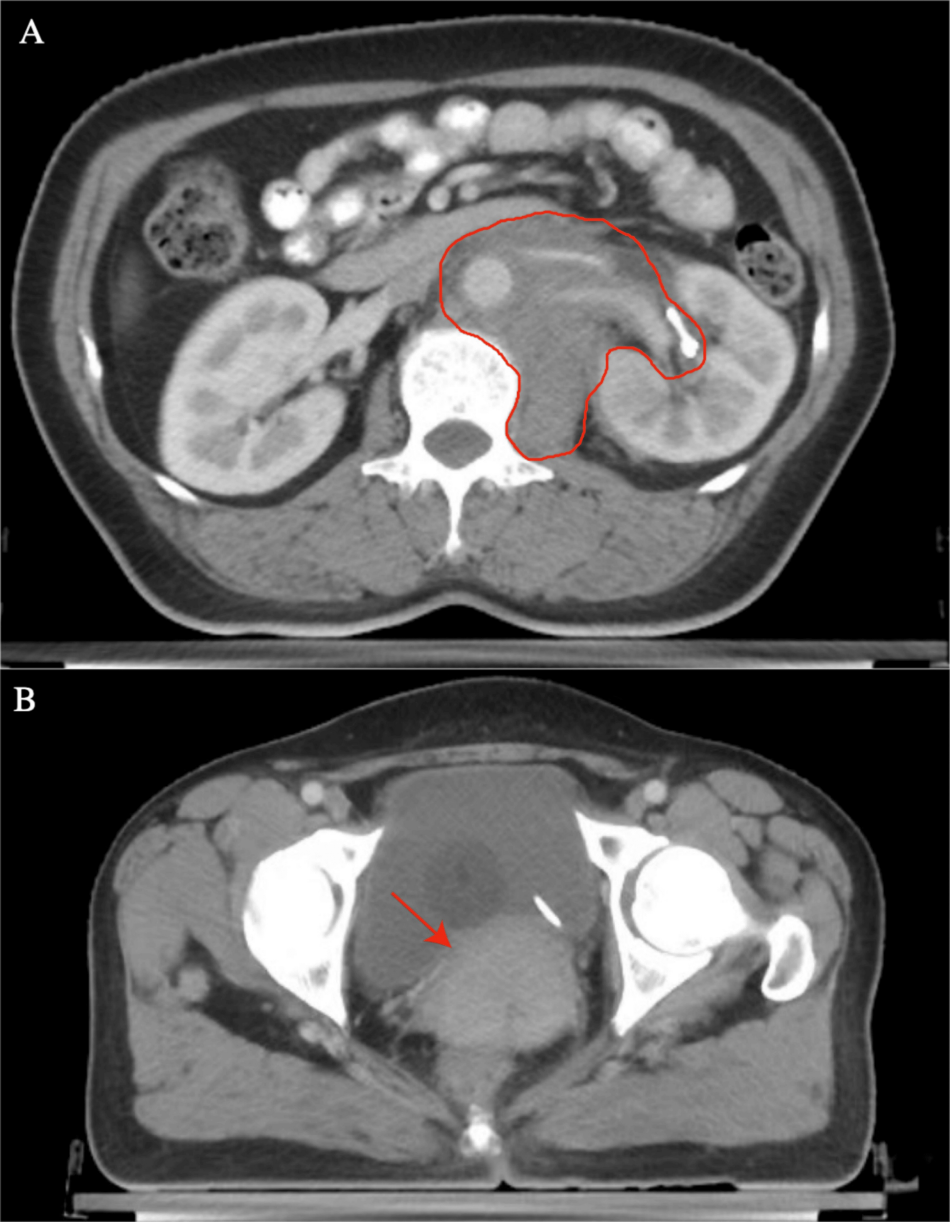
Enhanced computed tomography scan of the patient's abdomen and pelvis at the time of her initial diagnosis with granulocytic sarcoma. (A) An irregular soft tissue mass (outlined by the red line) is observed surrounding the left renal hilum, encasing the left renal hilum structures and the abdominal aorta, with indistinct borders between the mass and the left psoas muscle. (B) A 57×52 mm mass (indicated by the red arrow), with significant localized enhancement on the enhanced scan, is adjacent to the enlarged uterine cervix.

An enhanced computed tomography (CT) scan of the abdomen and pelvis revealed a retroperitoneal mass, an enlarged uterine cervix, and a 57×52 mm mass in the pelvic region (Figure 1).

The patient's laboratory results revealed several abnormalities. While her white blood cell count (10.8×10^9^/L) was mildly elevated, hemoglobin (133 g/L) and platelet counts (252×10^9^/L) were within normal limits. Liver and kidney function tests, including alanine aminotransferase (24 U/L), aspartate aminotransferase (28 U/L), total bilirubin (18.7 μmol/L), serum creatinine (71 μmol/L), and blood urea nitrogen (4.3 mmol/L), were all within reference ranges, indicating no significant hepatic or renal dysfunction at the time. Notably, the patient had hypokalemia (serum potassium 2.94 mmol/L) and hyponatremia (serum sodium 135 mmol/L). Additionally, serum chloride was also reduced (88 mmol/L). Blood glucose was significantly elevated at 18.58 mmol/L, suggesting poor glycemic control. Tumor markers, including carcinoembryonic antigen (1.22 ng/mL), cancer antigen 125 (13.8 U/mL), and cancer antigen 153 (14.8 U/mL), were all within normal limits. However, serum ferritin was elevated (284.76 ng/mL) (Table 1).

**Table 1:** Initial laboratory investigation results.

Date	Parameter	Value	Reference range
July 15, 2015	White blood cell	10.8×10^9^/L	3.5-9.5×10^9^/L
	Hemoglobin	133 g/L	115-150 g/L
	Platelet	252×10^9^/L	125-350×10^9^/L
	Alanine aminotransferase	24 U/L	7-40 U/L
	Aspartate aminotransferase	28 U/L	13-35 U/L
	Lactate dehydrogenase	238 U/L	110-250 U/L
	Total bilirubin	18.7 μmol/L	3-25 μmol/L
	Serum creatinine	71 μmol/L	35-80 μmol/L
	Blood urea nitrogen	4.3 mmol/L	2.9-7.1 mmol/L
	Serum albumin	38.7 g/L	40-55 g/L
	Serum potassium	2.94 mmol/L	3.50-5.30 mmol/L
	Serum sodium	135 mmol/L	137-147 mmol/L
	Serum chloride	88 mmol/L	99-110 mmol/L
	Blood glucose	18.58 mmol/L	3.90-6.10 mmol/L
	Carcinoembryonic antigen	1.22 ng/mL	0.00-6.50 ng/mL
	Cancer antigen 125	13.80 U/mL	0.00-35.00 U/mL
	Cancer antigen 153	14.80 U/mL	0.00-32.00 U/mL
	Serum ferritin	284.76 ng/mL	4.63-204.00 ng/mL

**Figure 2:**
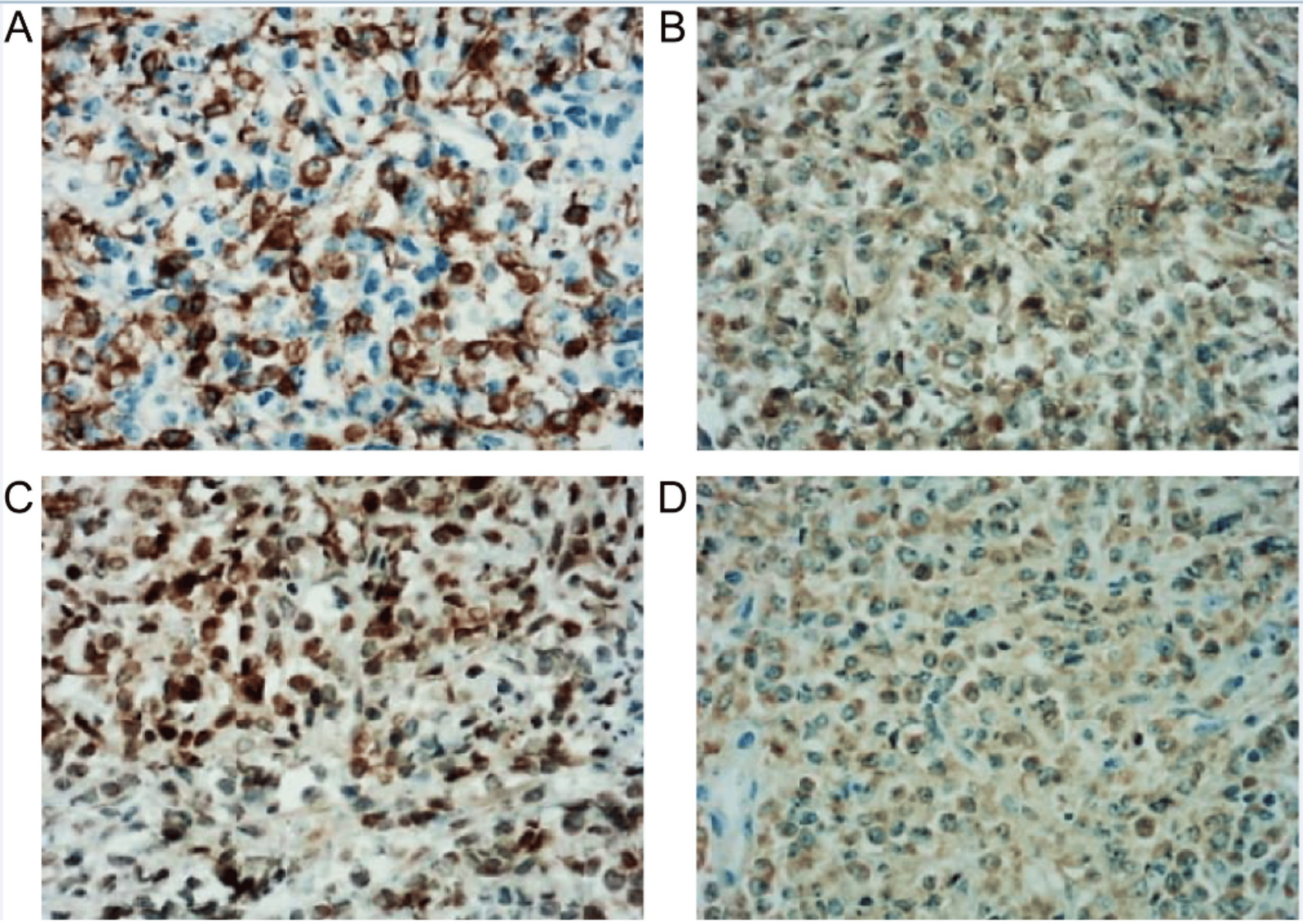
Immunohistochemical staining of the patient's cervical lesion. Neoplastic cells were positive for CD15 (A), CD33 (B), lysozyme (C), and myeloperoxidase (D). Original magnification: 400×.

Due to significant hydronephrosis caused by the mass, a double J ureteral catheter was placed via ureteroscopy. A biopsy of the cervical mass confirmed a diagnosis of GS. Microscopic sections show a monotonous infiltrate of abnormal cells, with mildly abundant cytoplasm. Neoplastic cells appear to be CD34+ and mark diffusely with myeloid and/or monocytic associated markers, with immunohistochemical (IHC) staining showing neoplastic cells positive for CD34, CD15, CD33, CD43, lysozyme, and myeloperoxidase (Figure 2).

**Figure 3:**
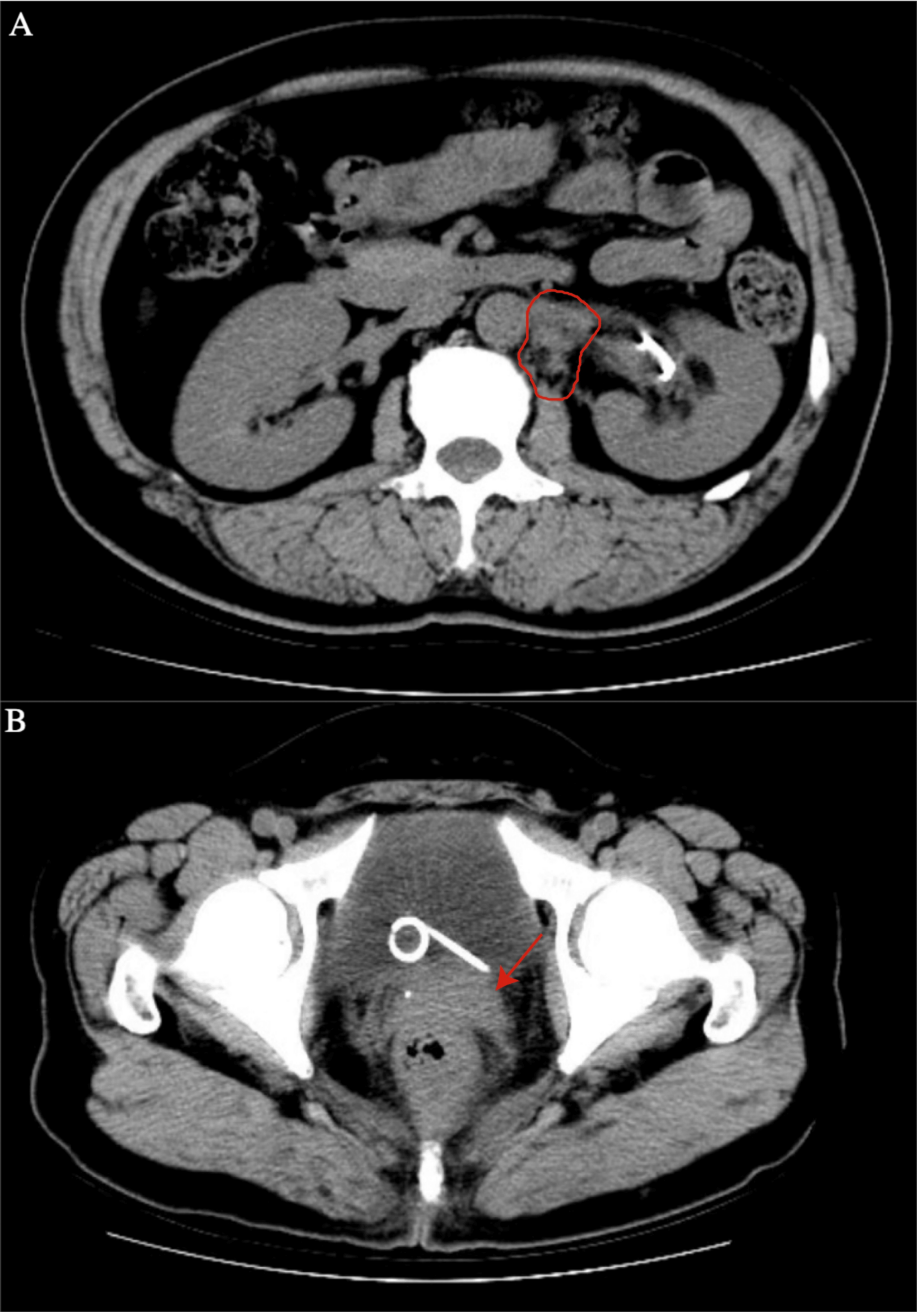
Enhanced computed tomography scan of the abdomen and pelvis following two cycles of idarubicin and cytarabine-based chemotherapy, showing a significant reduction in the previous mass. (A) Blurred fat planes are seen surrounding the left renal hilum and upper ureter, with a mass (outlined by the red line) visible at the level of the left renal hilum in the retroperitoneum. The mass shows poorly defined borders with the abdominal aorta, left renal artery, and left psoas muscle. (B) The cervix appears enlarged, with slightly obscured fat planes in the surrounding tissue (indicated by the red arrow).

Bone marrow aspiration and trephine biopsy showed no evidence of acute leukemia involvement. The patient was started on a chemotherapy regimen of idarubicin and cytarabine. After two cycles, a marked reduction in the size of the lesions was observed (Figure 3).

Repeated peripheral blood smears and bone marrow aspirations continued to show no signs of leukemia. Unfortunately, the patient's response to subsequent chemotherapy cycles (Table 2) was poor. She experienced complications, including bone marrow suppression, leading to recurrent infections and cerebral hemorrhage. The patient passed away in April 2016 due to these complications.

**Table 2:** The patient's chemotherapy regimen. VDS: vindesine; CTX: cyclophosphamide; IDA: idarubicin hydrochloride; PDN: prednisone; IFO: ifosfamide; THP: pirarubicin; d: day

Date	Regimen			
August 13, 2015	IDA 10 mg d1-3		Cytarabine 150 mg d1-3	
September 4, 2015	IDA 10 mg d1-3		Cytarabine 150 mg d1-3	
September 27, 2015	Cytarabine 3 g d1-3			
October 24, 2015	IDA 10 mg d1-3		Cytarabine 150 mg d1-3	
December 9, 2015	VDS 4 mg d1	CTX 0.6 mg d1	IDA 10 mg d1-3	PDN 60 mg d1-5
January 6, 2016	VDS 4 mg d1	CTX 0.6 mg d1	IDA 10 mg d1-3	PDN 60 mg d1-5
March 12, 2016	IFO 2 g d1	THP 80 mg d1	VDS 4 mg d1	PDN 100 mg d1-5

An enhanced CT scan of the abdomen and pelvis revealed a retroperitoneal mass, an enlarged uterine cervix, and a 57×52 mm mass in the pelvic region (Figure 1). The rarity of GS without concurrent acute leukemia is notable. Our systematic review employed specific search terms, namely, "cervical", "uterus cervical", "granulocytic sarcoma", and "chloroma". Ultimately, 35 relevant studies encompassing 42 participants were identified, restricted to those published in PubMed (https://www.ncbi.nlm.nih.gov/pubmed) (Table 3).

**Table 3:** Forty-two additional granulocytic sarcoma cases published in PubMed. O: operation; R: radiation; C: chemotherapy; NA: not available; FIGO: International Federation of Gynecology and Obstetrics; AML: acute myeloid leukemia; BSO: bilateral salpingo-oophorectomy; H: hysterectomy

Author	Published year	Country	Age (years)	Symptoms	Physical exam	Previous leukemia	Cytogenetics	FIGO stage	Treatment	Outcome	Survival
Chorlton et al. [7]	1974	USA	44	Vaginal bleeding	Cervical mass	NA	NA	Ib	BSO, H	Dead	3 months
Chorlton et al. [7]	1974	USA	75	Vaginal bleeding	Uterine tumor	NA	NA	IV	R, C	Dead	2 months
Seo et al. [8]	1977	USA	65	Vaginal bleeding	Cervical mass	No	NA	IIb	R, O, C	Dead	31 months
Kapadia et al. [9]	1978	USA	58	Vaginal bleeding	Cervical mass	No	NA	NA	C	Dead	8 months
Park et al. [10]	1980	Korea	36	Vaginal bleeding	Cervical erosion	No	NA	NA	O	NA	NA
Spahr et al. [11]	1982	NA	39	Abdominal pain, fever	Uterine tumor	No	NA	NA	None	Dead	6 days
Abeler et al. [12]	1983	Norway	35	Groin tumor, leg pain	Cervical mass	No	NA	NA	None	Dead	8 days
Abeler et al. [12]	1983	Norway	59	Vaginal bleeding	Cervical mass	NA	NA	NA	R	Dead	5 months
Harris and Scully [13]	1984	USA	48	Vaginal bleeding	Uterine tumor	NA	NA	NA	O, R	Dead	7 weeks
Harris and Scully [13]	1984	USA	71	Vaginal bleeding	Cervical mass	NA	NA	NA	C, R	NA	NA
Steinbock et al. [14]	1986	USA	53	Abdominal pain, vaginal bleeding, oliguria	Vaginal mass	AML	NA	NA	R, C	Dead	19 months
Banik et al. [15]	1989	UK	32	Vaginal bleeding	Cervical mass	No	Normal	NA	C	Alive	11 months
Zutter and Gersell [16]	1990	USA	36	Weight loss, fever, Bell's palsy	Cervical mass	Yes	NA	NA	C, R	Dead	36 months
Friedman et al. [17]	1992	USA	51	Menometrorrhagia and fatigue	Cervical mass	AML	NA	NA	C	Alive	13 months
Reynaud et al. [18]	1995	France	41	Vaginal bleeding	Cervical mass	No	NA	NA	NA	Dead	<2 months
Reynaud et al. [18]	1995	France	48	Vaginal bleeding	Waxy, green cervix	No	NA	NA	NA	Dead	24 months
Huter et al. [19]	1996	Austria	20	Vaginal bleeding	Cervical mass	No	NA	NA	C, R	Dead	3 years
Kamble et al. [20]	1997	India	33	Abdominal pain, fever	Cervical mass	No	NA	NA	None	Dead	<1 months
Delaflor-Weiss et al. [21]	1999	USA	67	Abdominal pain	Ulcerated mass	AML	NA	NA	C	Dead	13 months
Hernández et al. [22]	2002	Spain	45	Vaginal bleeding	Cervical mass	No	NA	NA	O, C	Dead	10 months
Hernández et al. [22]	2002	Spain	48	Vaginal pain	Vaginal mass	No	The cytogenetic study revealed a complex karyotype: 47, XX, t(1;4)(p34;q31), del(1)(p13), t(2;11)(p23;q13), del(5)(q12;q23), t(6;6)(p25;q15), +8[16]/46, XX[4]	NA	O, C	Dead	10 months
Maeng et al. [23]	2004	South Korea	30	Vaginal bleeding	Cervical mass	Yes	AML1/ETO (+)	NA	C, R	Alive	6 months
Lee et al. [24]	2004	South Korea	30	Vaginal bleeding	Cervical mass	Yes	NA	NA	C, R	Alive	8 months
Pathak et al. [25]	2005	Canada	33	Vaginal bleeding and pain	Pelvic mass	AML	NA	NA	C	Alive	7 months
Garcia et al. [26]	2006	USA	34	Dysmenorrhea	NA	No	NA	NA	C	Alive	12.5 years
Garcia et al. [26]	2006	USA	37	Vaginal bleeding	NA	No	NA	NA	C	Alive	2 months
Garcia et al. [26]	2006	USA	43	Vaginal bleeding	NA	No	NA	NA	NA	Alive	31 years
Pitz et al. [27]	2006	Canada	50	Vaginal bleeding	NA	AML	Normal	NA	C	Dead	11 months
Pullarkat et al. [28]	2007	USA	49	Right flank pain	Uterine mass	No	Normal	NA	C	NA	NA
Ko et al. [29]	2007	South Korea	50	Lower abdominal pain and right flank pain	A hard, tender mass in the pelvic cavity	CML	NA	NA	C	NA	NA
Gregor et al. [30]	2008	Czech Republic	61	Vaginal bleeding	The cervix was hypertrophic and deformed	AML	NA	NA	C	NA	NA
Henes et al. [31]	2009	Germany	81	Bleeding	-	AML	NA	NA	C	Dead	2 days
Kim et al. [32]	2010	France	30	Vaginal bleeding	Enlarged cervix	NA	Normal	NA	C	NA	NA
Chiang and Chen [33]	2010	China	51	Vaginal bleeding	Cervical mass	No	NA	NA	C	Dead	11 years
Ouansafi et al. [34]	2011	USA	50	Vaginal bleeding	Cervical lesion	No	t(11;19)(q23;p13.1) and a partial deletion of the short arm of chromosome 7	NA	O	NA	NA
Gill et al. [35]	2012	China	36	Vaginal bleeding	Lesion at the posterior cervical lip	NA	NA	NA	C	NA	NA
John et al. [36]	2013	USA	39	Lower quadrant abdominal pain, nausea, vomiting	Lower abdominal tenderness	No	Inversion of chromosome 16 (inv(16)(p13;q22))	NA	C	NA	NA
Ucar and Guryildirim [37]	2014	Turkey	23	Vaginal bleeding	Enlarged uterus	AML	NA	NA	C	NA	NA
Zheng et al. [38]	2015	China	51	Vaginal bleeding	Cervical mass	No	NA	NA	NA	NA	NA
Yamane et al. [39]	2017	Japan	29	Vaginal bleeding	Cervical mass	No	NA	NA	C	NA	NA
Capote et al. [40]	2018	Spain	35	Vaginal bleeding	Pelvic mass	No	NA	NA	O, C	NA	NA
Ye and Jiang [41]	2022	China	45	Vaginal bleeding	Enlarged cervix	NA	45,X,-X, t(1;4)(q21;q25)2/46, XX18	NA	O, C	NA	NA

## Discussion

GS without concurrent acute leukemia is an uncommon presentation, particularly within the female reproductive system. In our review of 42 cases, vaginal bleeding emerged as the most frequent symptom, often leading to the identification of cervical masses. Despite the variability in clinical presentations, such as abdominal pain, fever, and menstrual irregularities, all cases involved a detectable mass through imaging or physical examination, similar to our patient, who presented with a cervical mass and hydronephrosis.

Histologically, GS is composed of immature myeloid cells, characterized by round or oval nuclei, one or two nucleoli, and eosinophilic cytoplasm [9]. Unlike other malignancies, GS often presents with distinct coloration, texture, or morphology at the tumor site. Clinical features may include fever, skin lesions, localized pain, and elevated eosinophil or leukemic cell counts in the peripheral blood [42,43]. Due to its morphological similarity to histiocytic lymphoma, diagnosing GS can be challenging [9,10]. Histopathological confirmation is critical, typically relying on positive staining for markers such as myeloperoxidase, CD34, CD15, CD43, and Ki67, while markers like L26, TdT, and CD10 are negative. This diagnostic process helps differentiate GS from other tumors, such as lymphomas, leukemias, myelodysplastic syndromes, and metastatic lesions [44].

Kei et al. reported a rare case of GS with infiltration of the corpus callosum, highlighting the need to consider uncommon pathologies in lesions affecting this region. F-18 fluorodeoxyglucose (FDG) PET scans have proven useful in detecting and monitoring GS [45]. Similarly, Harris and Scully demonstrated the utility of MRI in diagnosing spinal GS, with features including multiple spinal masses, nerve root thickening, sclerotic lesions, and diffuse bone marrow infiltration [13].

The clinical manifestations of GS vary and may include extramedullary masses, cough, dyspnea, nasal congestion, headache, bleeding, anemia, and pain [46]. In patients with cervical GS, vaginal or postcoital bleeding and abdominal discomfort and systemic symptoms are commonly reported [25]. In our case, the patient initially presented with vaginal bleeding, but no significant abnormalities were noted on physical examination.

A retrospective review of untreated GS patients at the MD Anderson Cancer Center from 1990 to 2002 showed that 65% of non-leukemic GS cases achieved complete remission and 5% partial remission and the remainder did not undergo treatment. Patients with abnormal chromosome 8 had lower survival rates compared to those with normal karyotypes, underscoring the need for novel therapeutic strategies [46]. Emerging genetic mutations associated with GS prognosis also warrant further investigation, as they could inform personalized treatment approaches.

Isolated GS has been associated with relatively favorable prognoses, although differentiating it from GS with bone marrow involvement can be challenging. For patients with solitary GS, systemic chemotherapy or autologous/allogeneic bone marrow transplantation significantly improves long-term outcomes [47]. Patients undergoing bone marrow transplantation had a 76% survival rate at 48 months, compared to 0% for those who did not receive transplantation [48].

Seo et al. [8] reported a case of cervical GS developing into a leukemia-like syndrome postoperatively, raising questions about whether surgery might accelerate disease progression. Paydas et al. [49] described a patient who developed complications after tibial GS surgery, ultimately requiring amputation and subsequently developing AML. Conversely, Bakst et al. demonstrated that radiotherapy effectively controlled local GS and alleviated symptoms without causing severe side effects [50].

Several studies have highlighted improved disease-free survival rates in GS patients who received systemic chemotherapy, compared to those treated primarily with local therapies such as radiation or surgery. A study by Tsimberidou et al. [46] emphasized the importance of chemotherapy for extending survival, while stem cell transplantation was found to be crucial for long-term outcomes. A 2007 study [48] reported a 76% overall survival (OS) rate at 48 months in transplanted patients compared to 0% in non-transplanted patients. Another study in 2008 demonstrated a five-year OS rate of 47% and event-free survival of 36% in patients who underwent allogeneic stem cell transplantation for GS. Patients older than 15 years and those achieving remission prior to transplantation had the most favorable outcomes [51].

Targeted therapies, including CD33 [52] monoclonal antibodies, have shown potential, with some patients achieving partial remission, while FIP1L1-PDGFRA gene-positive cases achieved complete remission with tyrosine kinase inhibitors such as imatinib [53]. Collectively, these findings underscore the importance of systemic chemotherapy, radiotherapy, surgical intervention, and stem cell transplantation in the treatment of GS, with targeted therapy representing a promising new frontier. 

Managing GS requires a multidisciplinary approach including surgery, radiotherapy, chemotherapy, and targeted therapies. Early diagnosis and multimodal treatment strategies are critical for improving survival outcomes. Historically, GS prognosis was poor, but advancements in diagnostic techniques and treatment modalities have extended survival for many patients [54]. In this case, the patient received chemotherapy due to the availability of CD33 monoclonal antibody and allogeneic hematopoietic stem cell transplantation (allo-HSCT), but unfortunately, survival was limited to nine months.

This study has several limitations. First, due to the rarity of GS in the cervix, our analysis is limited by the small sample size and reliance on case reports and retrospective studies. Second, the lack of uniformity in treatment protocols across the cases reviewed makes it difficult to draw definitive conclusions regarding the optimal management strategies for cervical GS. Finally, genetic data were not consistently available for all cases, which limits our ability to fully explore the potential role of genetic mutations in disease prognosis and treatment outcomes.

## Conclusions

This report presents a rare case of GS of the cervix without concurrent acute leukemia. While the patient initially responded to chemotherapy, subsequent treatment cycles were ineffective, and the disease progressed, leading to fatal complications. Among the cases reviewed, outcomes were generally poor, with most patients succumbing to the disease within months. This parallels the outcome in our case, where the patient initially responded to chemotherapy but ultimately developed complications, including bone marrow suppression, recurrent infections, and cerebral hemorrhage. This highlights the aggressive nature of GS in the cervix. The case reinforces the importance of early diagnosis, thorough histopathological evaluation, and the exploration of personalized treatment strategies, particularly in GS cases without associated leukemia. Further research is needed to investigate novel therapeutic agents and targeted treatments to improve outcomes for patients with this rare malignancy.
